# Care delivery team composition effect on hospitalization risk in African Americans with congestive heart failure

**DOI:** 10.1371/journal.pone.0286363

**Published:** 2023-06-15

**Authors:** Tremaine B. Williams, Alisha Crump, Maryam Y. Garza, Nadia Parker, Simeon Simmons, Riley Lipschitz, Kevin Wayne Sexton

**Affiliations:** 1 Department of Biomedical Informatics, University of Arkansas for Medical Sciences, Little Rock, Arkansas, United States of America; 2 Department of Epidemiology, University of Arkansas for Medical Sciences, Little Rock, Arkansas, United States of America; 3 Department of Internal Medicine, University of Arkansas for Medical Sciences, Little Rock, Arkansas, United States of America; 4 Department of Surgery, University of Arkansas for Medical Sciences, Little Rock, Arkansas, United States of America; 5 Department of Health Policy and Management, University of Arkansas for Medical Sciences, Little Rock, Arkansas, United States of America; Madras Medical College, INDIA

## Abstract

The care delivery team (CDT) is critical to providing care access and equity to patients who are disproportionately impacted by congestive heart failure (CHF). However, the specific clinical roles that are associated with care outcomes are unknown. The objective of this study was to examine the extent to which specific clinical roles within CDTs were associated with care outcomes in African Americans (AA) with CHF. Deidentified electronic medical record data were collected on 5,962 patients, representing 80,921 care encounters with 3,284 clinicians between January 1, 2014 and December 31, 2021. Binomial logistic regression assessed associations of specific clinical roles and the Mann Whitney-U assessed racial differences in outcomes. AAs accounted for only 26% of the study population but generated 48% of total care encounters, the same percentage of care encounters generated by the largest racial group (i.e., Caucasian Americans; 69% of the study population). AAs had a significantly higher number of hospitalizations and readmissions than Caucasian Americans. However, AAs had a significantly higher number of days at home and significantly lower care charges than Caucasian Americans. Among all CHF patients, patients with a Registered Nurse on their CDT were less likely to have a hospitalization (i.e. 30%) and a high number of readmissions (i.e., 31%) during the 7-year study period. When stratified by heart failure phenotype, the most severe patients who had a Registered Nurse on their CDT were 88% less likely to have a hospitalization and 50% less likely to have a high number of readmissions. Similar decreases in the likelihood of hospitalization and readmission were also found in less severe cases of heart failure. Specific clinical roles are associated with CHF care outcomes. Consideration must be given to developing and testing the efficacy of more specialized, empirical models of CDT composition to reduce the disproportionate impact of CHF.

## Background and significance

Despite rapid pharmacologic and therapeutic advancements in congestive heart failure (CHF) care, racial disparities in CHF care outcomes persist as a national problem [1, 2]. By 2030, CHF prevalence is expected to exceed 8 million, disproportionately impacting African Americans (i.e., 2.2 times greater prevalence than Caucasian Americans) [1–3]. The estimated $69 billion in increased care costs will devastate patients, oversaturating care delivery systems and driving medical cost-related poverty in rural areas of states such as Arkansas where in some counties more than 35% of African Americans (AA) are already living in poverty [1–5]. AAs have higher CHF prevalence, increased hospitalization risk, and higher 5-year mortality risk after initial CHF diagnosis than Caucasian Americans (CA) [4–12]. Yet; these risks are not attributed to genetic differences, given that patients are 99.9% similar with no variation between racial groups [1, 6–11]. Since genetics are not attributed, there is a need to target other factors with the potential to impact care outcomes.

Arkansas is uniquely positioned to serve as a model for assessing more innovative solutions to addressing health disparities, considering CHF prevalence and mortality risk in some Arkansas counties are nearing twice the national average [3–5]. To alleviate the current and estimated disproportionate impact of CHF on AAs, [1] more comprehensive solutions (i.e., non-pharmacologic, non-therapeutic) are needed [12–17]. This need has motivated sociocultural and environmental explorations into how the care delivery team (CDT) influences the risk of improved care outcomes (i.e., decreased hospitalizations, readmissions, and charges; increased days between readmissions) and how this influence can be factored into risk stratification of patients [18–24].

**Fig 1:**
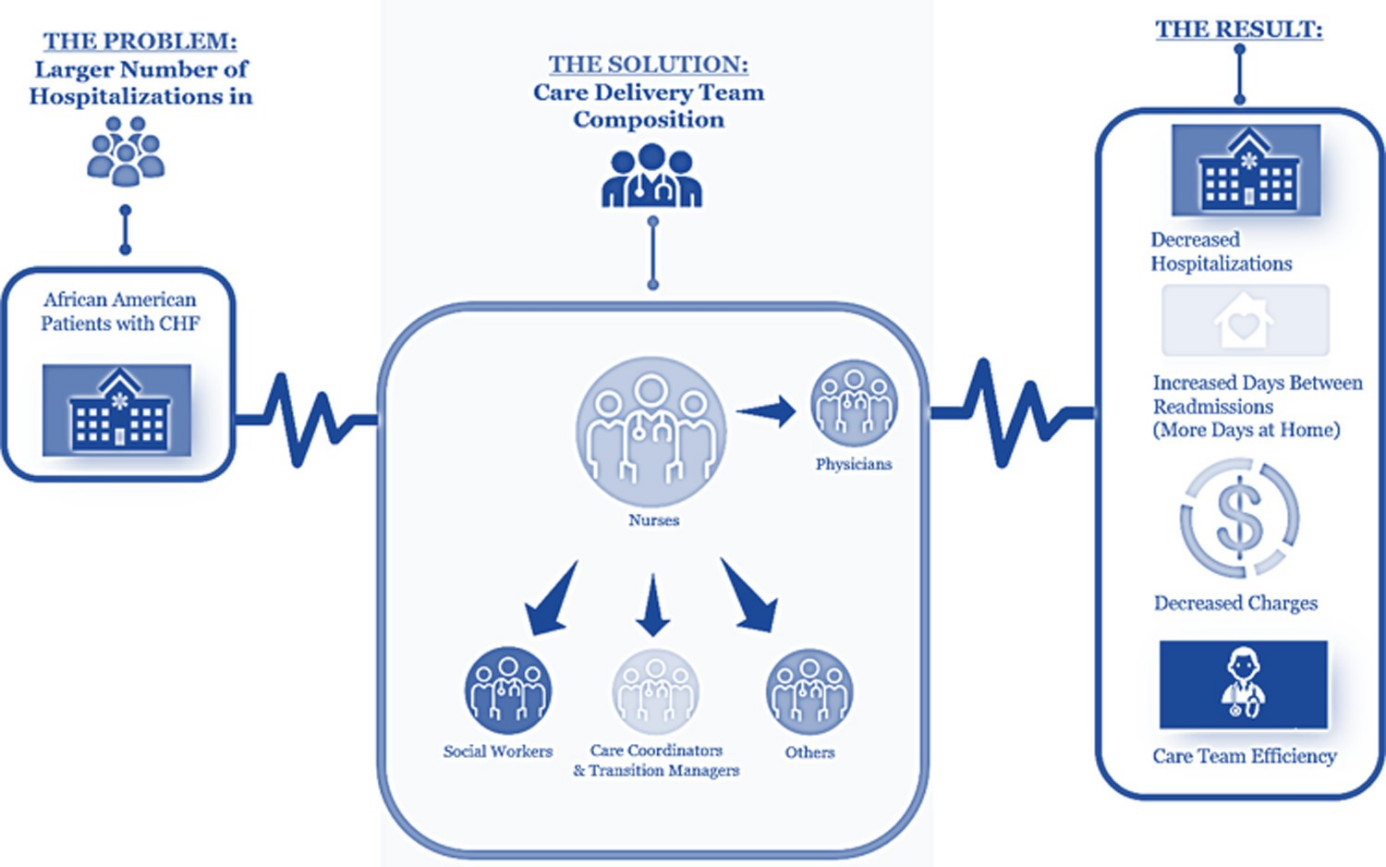
Problem, solution, and results.

Risk stratification alleviates the disproportionate impact of AA patients with CHF by allowing CDTs to better prioritize the needs of their patient populations [13–25]. Yet, current risk stratification approaches are limited by linearly focusing on patient-level risk factors, [16, 17, 26–43] excluding group [CDT] and organizational-level [clinic/hospital] factors of high-quality care delivery that have been associated with improved outcomes in patients with cardiovascular diseases [18–25]. However, only fragmented evidence of how to optimally compose the CDT exists [22, 23]. Defining the optimal CDT composition is particularly impactful in the resource-constrained, rural hospitals of Arkansas and similar states, [44, 45] in which clinical workforce shortages fuel racial disparities in care access and increase the risk of adverse cardiovascular events in AA patients with CHF [3–5, 44, 46, 47]. Thus, the objective of this study was to examine the extent to which specific clinical roles within CDTs were associated with improved care outcomes of AA patients with CHF [Fig 1].

## Materials and methods

### Data collection and study procedures

**Fig 2:**
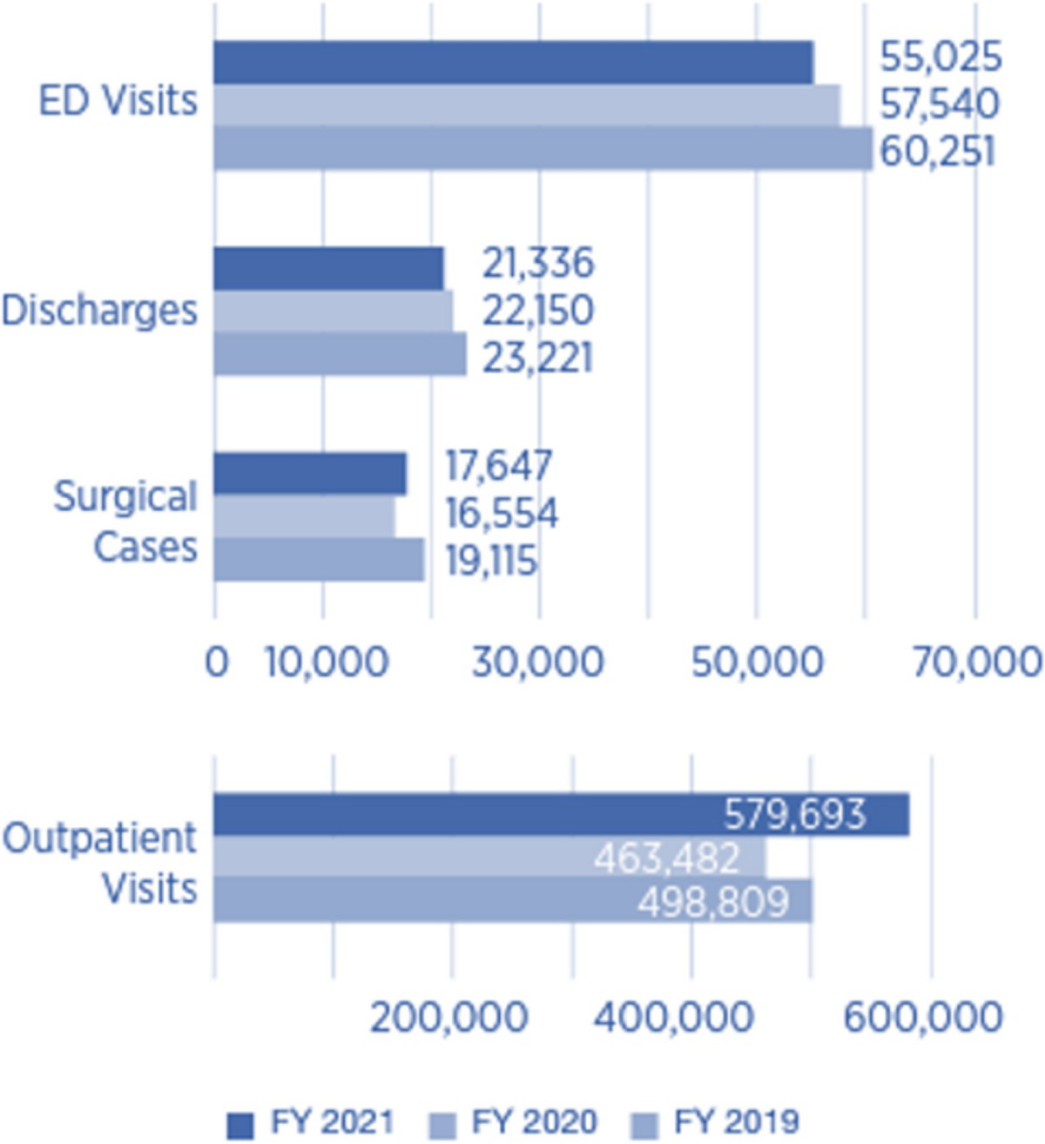
UAMS patient care utilization.

A retrospective, observational study design was used to extract deidentified EMR data from the Arkansas Clinical Data Repository (AR-CDR) [48] on all CHF patients who received care at the University of Arkansas for Medical Sciences (UAMS) between January 1, 2014 and December 31, 2021. The EMR has proven to be a rich source of data for understanding care delivery teams and their impact on the care outcomes of patients with cardiovascular and other chronic diseases [18, 48]. UAMS is Arkansas’ only academic medical center and only Level One Trauma Center. The AR-CDR contains data on patient care encounters at UAMS’ main campus in Little Rock, AR, a 535-bed hospital (i.e., 431 adult beds, 64 newborn bassinets, 40 psychiatry beds) [49]. The AR-CDR also contains data on over 30 of UAMS’ regional clinics in rural and metropolitan areas of Northeast, Northwest, Central, Southeast, and Southwest Arkansas. Fig 2 reflects annual patient care utilization at UAMS [49]. Eligibility criteria limited subjects to patients who were aged 18–105 with CHF. CHF diagnosis was identified using the International Classification of Diseases *Rv*.10 (ICD-10) codes. The study procedures (Protocol number: 262593) were approved by the Institutional Review Board at UAMS.

### Study variables

The AR-CDR data contained all patient, CDT, and outcome variables. Patient variables included deidentified patient ID, age, sex, race, ethnicity, clinical diagnosis, comorbidities, medical history, medications, and heart failure phenotype data. Care outcome variables included the number of hospitalizations (i.e., patient admitted to the hospital without an admission within the preceding 30 days), the number of readmissions (i.e., patient admitted to the hospital within 30 days of a hospital discharge), the number of days between readmissions, and the high charge care encounter (i.e., the amount of dollars billed to the patient by the hospital for medical care rendered). All patient encounter dates including primary care, hospitalizations, and follow-up periods for readmission were based on the date of first entry in the EMR (i.e., the date of care). Time lapses between initial hospitalization and readmission varied between patients due to factors including patient’s need for care. CDT variables, the AR-CDR data contained an edge list that associated patient encounters with the specific clinicians (i.e., clinical roles) who provided care during each encounter. The AR-CDR data indicated a total of up to 60 clinical roles on the CDTs of CHF patients. However, only clinical roles that have been associated with hospitalizations, readmissions, days between readmissions, and high charge encounters were included: physician, resident, advanced nurse practitioner, registered nurse, licensed practical nurse, patient care technician, care manager, social worker, physical therapist, and occupational therapist.

### Statistical analysis

Binomial logistic regression was used to ascertain the effects of age, sex, race, ethnicity, and CDT composition on the likelihood that participants had a hospitalization, a high number of readmissions, a high number of days between readmissions, and a high charge care encounter. Within the regressions, odd ratios represented associations between variables. All continuous care outcome variables and age were dichotomized at the median to distinguish a high/low threshold, supporting the dichotomy assumption of the binomial logistic regression. For example; a “high charge encounter” was defined as a charge above the median charge for a care encounter (i.e., $22,403) and was transformed to “1”, representing “yes; high charge”, and a charge below the median was transformed to “0”, representing “No; low charge”. These transformations were replicated for hospitalizations, readmissions, and days between readmissions, and patient age. Furthermore, a Mann Whitney-U test was used to identify differences between AA and CA care outcomes. All patient, CDT, and care outcome variables were treated as continuous and mutually exclusive within the Mann Whitney-U analysis.

## Results

The study identified a total of 5,962 CHF patients who met the criteria. These patients generated a total of 80,921 care encounters during the study period. Table 1 provides demographic information on the study population, stratified by patients and their encounters. CAs were the largest racial group, accounting for 69% (i.e., 4,139) of patients and generating 48% (i.e., 39,187) of total care encounters. AAs were the second largest racial group, accounting for 26% (i.e., 1,576) of patients and generating the same percentage of care encounters as CAs (i.e., 48%; 39,076). Native Hawaiian and other Pacific Islanders were the smallest racial group, accounting for less than 1% (i.e., 5) of patients, generating less than 1% (i.e., 26) of care encounters. Patients of Hispanic, Latin, and Spanish ethnicity accounted for 1% (i.e., 74) of patients and generated approximately 2% (i.e., 1,892) of care encounters.

**Table 1:** Demographics stratified by total patients and their encounters.

Demographic Variables	Patients (n = 5,962)	Total care encounters (n = 80,921)
Median age (Std. Dev.)	59 (15.2)	
**Race, number (%)**		
Caucasian American	4,139 (0.69)	39,187 (0.48)
African American	1,576 (0.26)	39,076 (0.48)
Asian American	20 (<0.01)	185 (<0.01)
American Indian/Alaskan Native	7 (<0.01)	249 (<0.01)
Native Hawaiian/Other Pacific Islander	5 (<0.01)	26 (<0.01)
Other race	153 (0.03)	2,084 (0.03)
Unknown race	62 (0.01)	114 (<0.01)
**Ethnicity**, Hispanic/Latin/Spanish	74 (0.01)	1,892 (0.02)
**Sex, number (%)**		
Male	3,036 (0.51)	37,484 (0.46)
Female	2,926 (0.49)	43,437 (0.54)

Of the 80,921 care encounters, 26,795 (i.e., 33%) were primary care encounters and 54,126 (i.e., 67%) were hospitalization encounters. Table 2 provides demographic information on these primary care and hospitalization encounters. When further stratified, CAs were the largest racial group in both primary care and hospitalization encounters, accounting for 57% (i.e., 435) and 71% (i.e., 3,703), respectively. AAs were the second largest racial group in both primary care and hospitalization encounters, accounting for 40% (i.e., 307) and 24% (i.e., 1,269), respectively. AAs, as the second largest racial group in primary care encounters, generated 16,282 (i.e., 61%) primary care encounters, nearly twice the number of primary care encounters as CAs, the largest racial group (i.e., 35%; 9,398). Notably, AA patients generated 42% (i.e., 22,794) of hospitalization encounters and were only 24% (i.e., 1,269) of the patient population.

**Table 2:** Demographics stratified by primary care and hospitalization encounters.

Demographic Variables	Total Patients (n = 5,962) and Care Encounters (n = 80,921)			
	Primary care encounters		Hospitalization encounters	
	Patients (n = 764)	Primary care encounters (n = 26,795)	Patients (n = 5,198)	Hospitalization encounters (n = 54,126)
Age, median (Std. Dev.)	60 (15.2)		66 (14.79)	
**Race, number (%)**				
Caucasian American	435 (0.57)	9,398 (0.35)	3,703 (0.71)	29,789 (0.55)
African American	307 (0.40)	16,282 (0.61)	1,269 (0.24)	22,794 (0.42)
Asian American	1 (<0.01)	25 (<0.01)	19 (<0.01)	160 (<0.01)
American Indian/Alaskan Native	1 (<0.01)	87 (<0.01)	6 (<0.01)	162 (<0.01)
Native Hawaiian/Other Pacific Islander	1 (<0.01)	9 (<0.01)	5 (<0.01)	17 (<0.01)
Other Race	12 (0.02)	981 (0.04)	141 (0.03)	1103 (0.02)
Unknown Race	7 (0.01)	13 (<0.01)	55 (0.01)	101 (<0.01)
**Ethnicity**, Hispanic/Latin/Spanish	9 (0.01)	990 (0.04)	65 (0.01)	902 (0.02)
**Sex, number (%)**				
Male	376 (0.49)	12,082 (0.45)	2,660 (0.51)	25,402 (0.47)
Female	388 (0.51)	14,713 (0.55)	2,538 (0.49)	28,724 (0.53)

During the 80,921 care encounters, the 5,962 patients were provided care by 3,284 clinicians (Table 3). Table 3 illustrates clinician engagement with CHF patients. The largest number of clinical roles were registered nurses (i.e., 1,877) and the smallest was licensed practical nurses (i.e., 17). The median (Table 3) illustrates how less than half of CHF patient encounters did not involve care provided by a resident, advanced nurse practitioner, licensed practical nurse, patient care technician, care manager, social worker, physical therapist, or occupational therapist. However, 50% of CHF patients were cared for by at least one physician and at least three registered nurses.

**Table 3:** Patient CDT study demographics.

Clinical Roles	Number (%)	Mean (SD)	Median	Mode	Range
Physician	510 (16%)	0.71 (0.83)	1	0	0–15
Resident	334 (10%)	0.52 (0.67)	0	0	0–6
Advanced nurse practitioner	111 (3%)	0.35 (0.97)	0	0	0–27
Registered nurse	1,877 (57%)	6.97 (11.64)	3	1	0–58
Licensed practical nurse	17 (1%)	0.03 (0.70)	0	0	0–17
Patient care technician	266 (8%)	0.47 (2.10)	0	0	0–50
Care manager	63 (2%)	0.34 (0.80)	0	0	0–17
Social worker	42 (1%)	0.14 (0.49)	0	0	0–24
Physical therapist	39 (1%)	0.50 (1.35)	0	0	0–30
Occupational therapist	25 (1%)	0.28 (0.97)	0	0	0–30

**Fig 3:**
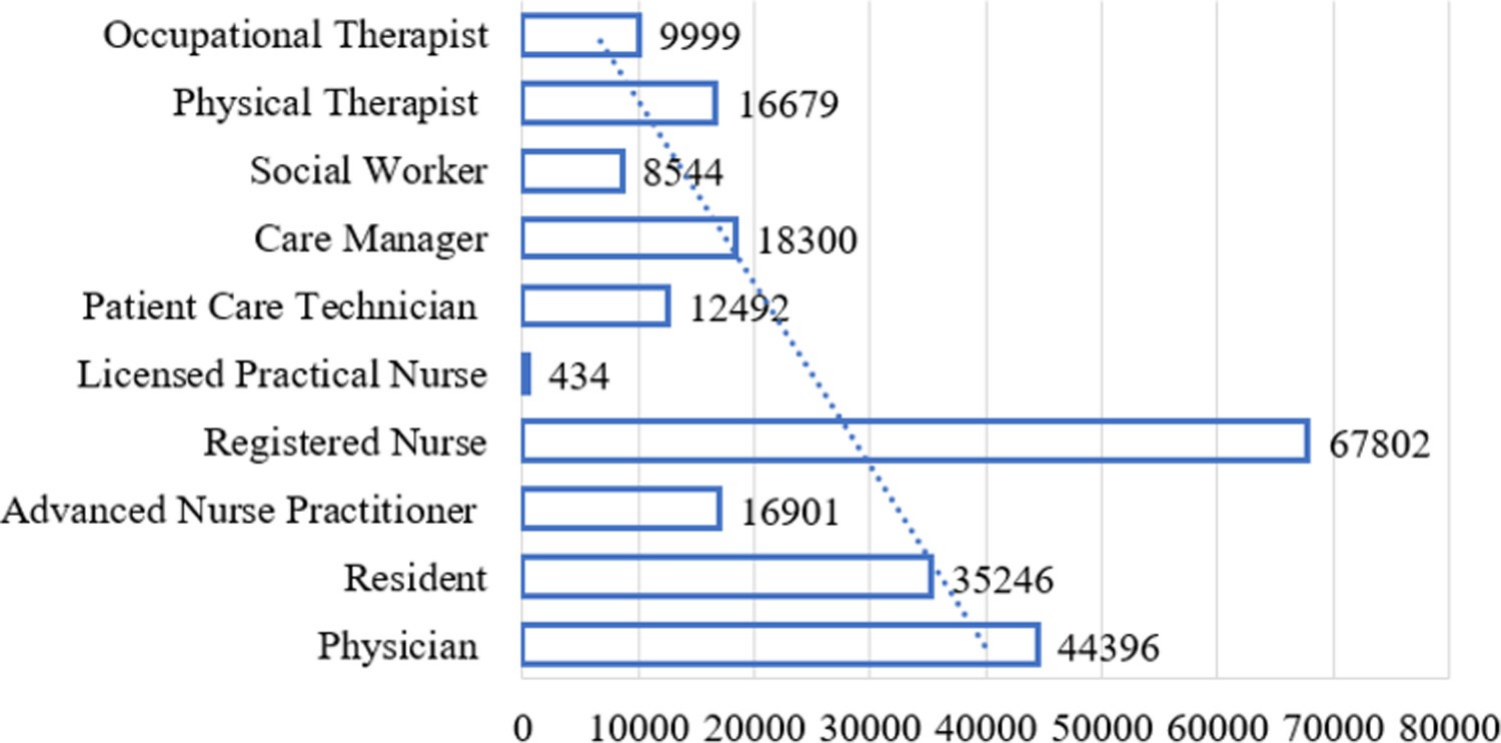
Total patient-clinician interactions stratified by clinical role.

Within the natural flow of clinical operations, the 3,284 clinicians may have provided care for the 5,962 patients more than once (Fig 3), randomly composing groups of clinicians (i.e., CDTs) based on hospital and clinic factors (e.g., staffing needs, availability of clinical staff to work, expertise, experience, and the specific care needs of patients who visited the clinic or hospital on any given day during the study period). However, the random nature of how the clinical teams were composed helped mitigate potential systematic concerns surrounding the consistent matching of specific patients with specific clinicians. Fig 3 reflects the number of times each of the 10 clinical roles (i.e., a clinician) delivered care by engaging with the 5,962 patients as a member of a patient’s CDT (i.e., a patient-clinician interaction). The 80,921 care encounters generated 230,793 patient-clinician interactions (Fig 3). Within these interactions, Registered Nurses were the most highly engaged clinical role in care delivery, as represented by the 29% (i.e., 67,802 instances) of total instances in which they provided care to the 5,962 CHF patients. Licensed Practical Nurses were the least engaged clinical role in care delivery (i.e., 434 instances; <1% of total instances).

Tables 4–7 present adjusted odds ratios for 4 binomial logistic regressions which reflect the effects of age, sex, race, ethnicity, and CDT composition on the likelihood that participants had a hospitalization (Model 1), a high number of readmissions (Model 2), a high number of days between readmissions (i.e., more days at home; Model 3), and a high charge hospitalization encounter (Model 4). All regression models were statistically significant (*p* = .001). To address potential confounding variables, odds ratios in Tables 5–7 stratified 1,664 of the study’s patients (i.e., who underwent an ethnographic examination at hospital admission) by heart failure phenotype. Tables 5–7 were additionally adjusted for medications, comorbidities, and patient medical history.

**Table 4:** Binomial logistic regression odds ratios of all patient, CDT, and outcome variables (n = 5,962).

Variables	Model 1: Hospitalization Odds ratio (95%CI)	Model 2: High number of readmissions Odds ratio (95%CI)	Model 3: High number of days between readmissions Odds ratio (95%CI)	Model 4: High charge encounter Odds ratio (95%CI)
Patient Variables**				
Age	0.97 (0.92–1.01)	0.98 (0.94–1.02)	1.09 (1.05–1.13)^a^	1.00 (0.96–1.04)
Male	0.98 (0.94–1.03)	1.11 (1.06–1.15)^a^	0.87 (0.84–0.90)^a^	1.04 (1.04–1.08)
Female	1	1	1	1
Caucasian American	0.31 (0.16–0.61)^a^	7.46 (3.26–17.04)^a^	5.52 (3.35–9.08)^a^	0.54 (0.33–0.89)^b^
African American	0.16 (0.08–0.31)^a^	11.20 (4.90–25.59)^a^	7.84 (4.76–12.90)^a^	0.34 (0.21–0.56)^a^
Hispanic/Latin/Spanish	0.38 (0.30–0.49)^a^	2.20 (1.78–2.71)^a^	1.01 (0.86–1.20)	0.57 (0.47–0.69)^a^
Asian American	0.70 (0.30–1.64)	11.56 (4.72–28.31)^a^	11.46 (6.33–20.76)^a^	0.82 (0.44–1.53)
American Indian/ Alaskan Native	0.26 (0.12–0.56)	10.11 (4.19–24.39)^a^	14.33 (8.10–25.35)^a^	0.42 (0.23–0.75)^b^
Native Hawaiian/Other Pacific Islander	0.15 (0.38–0.63)^b^	3.59 (0.78–16.62)	15.77 (5.63–44.14)^a^	0.37 (0.13–1.08)
Other Race	0.25 (0.12–0.51)^a^	13.83 (5.93–32.22)^a^	2.70 (1.61–4.54)^a^	0.44 (0.26–0.75)^b^
**CDT Composition**				
Physician	3.02 (2.86–3.19)^a^	0.87 (0.83–0.90)^a^	0.98 (0.95–1.02)	2.59 (2.48–2.71)^a^
Resident	1.04 (0.98–1.10)	0.87 (0.83–0.91)^a^	1.11 (1.07–1.15)^a^	1.30 (1.24–1.35)^a^
Advanced nurse practitioner	1.12 (1.05–1.19)^a^	0.96 (0.91–1.00)	0.89 (0.85–0.92)^a^	1.79 (1.71–1.87)^a^
Registered nurse	0.70 (0.66–0.76)^a^	0.69 (0.65–0.73)^a^	0.92 (0.88–0.97)^a^	1.06 (1.01–1.11)^b^
Licensed practical nurse	435276451 (0-∞)	0.60 (0.47–0.77)^a^	1.37 (1.10–1.69)^b^	0.88 (0.69–1.11)
Patient care technician	29.43 (25.89–33.45)^a^	0.81 (0.77–0.86)^a^	0.92 (0.88–0.96)^a^	3.77 (3.55–4.01)^a^
Care manager	21.87 (19.96–23.96)^a^	0.81 (0.77–0.85)^a^	0.96 (0.93–1.00)	4.84 (4.60–5.09)^a^
Social worker	1491 (480.14–4632.97)^a^	0.83 (0.78–0.89)^a^	0.88 (0.84–0.93)^a^	3.32 (3.08–3.59)^a^
Physical therapist	216.18 (141.97–329.16)^a^	0.72 (0.67–0.77)^a^	0.98 (0.80–0.90)^a^	8.82 (7.40–8.82)^a^
Occupational therapist	7.30 (4.12–12.96)^a^	0.86 (0.79–0.93)^a^	0.98 (0.91–1.05)	1.32 (1.17–1.48)^a^

*Significance:

^a^*P* < .001

^b^*P* < .05’

**Odds ratios for unknown race were not provided because they accounted for <1% of encounters and were not statistically significant.

**Table 5:** Binomial logistic regression odds ratios of HErEF patients (n = 628).

Variables	Model 1: Hospitalization Odds ratio (95%CI)	Model 2: High number of readmissions Odds ratio (95%CI)	Model 3: High number of days between readmissions Odds ratio (95%CI)	Model 4: High charge encounter Odds ratio (95%CI)
Patient Variables**				
Age	1.35 (0.85–2.15)	1.09 (0.79–1.49)	1.05 (0.82–1.35)	0.84 (0.63–1.17)
Male	1.35 (0.83–2.21)	0.87 (0.63–1.19)	1.13 (0.87–1.45)	1.14 (0.85–1.52)
Female	0	0	0	0
Caucasian American	0	7657607964 (0-∞)	3540804219 (0-∞)	0
African American	0	9699204213 (0-∞)	3482441437 (0-∞)	0
Hispanic/Latin/Spanish	0	183348636 (0-∞)	1.13 (0.27–4.75)	0.41 (0.08–2.25)
Asian American	0	441224098 (0-∞)	10111089576 (0-∞)	0
American Indian/ Alaskan Native	0	18602937 (0-∞)	47769543829 (0-∞)	0
Native Hawaiian/Other Pacific Islander	0	0	0	0
Other Race	0	85990409 (0-∞)	1477769545 (0-∞)	0
**Comorbidities and Medical History**				
Anemia deficiency	1.21 (0.73–2.03)	1.93 (1.40–2.66)^a^	0.71 (0.54–0.92)^a^	1.28 (0.94–1.73)
Coagulopathy	1.44 (0.73–2.86)	0.80 (0.53–1.21)	0.75 (0.54–1.03)	1.18 (0.81–1.71)
Diabetes (complicated)	0.79 (0.39–1.61)	2.91 (1.82–4.65)^a^	0.79 (0.55–1.13)	0.90 (0.59–1.36)
Diabetes (uncomplicated)	0.93 (0.45–1.61)	0.84 (0.54–1.31)	0.68 (0.48–0.96)^b^	0.96 (0.59–1.36)
Hypertension (complicated)	0	0.65 (0.06–7.71)	1.58 (0.29–8.52)	0.64 (0.12–3.56)
Metastatic cancer	0.39 (0.11–1.33)	5.66 (2.09–15.33)^a^	0.76 (0.38–1.49)	1.07 (0.49–2.33)
Obese	1.04 (0.64–1.70)	2.39 (1.74–3.27)^a^	1.12 (0.87–1.45)	1.00 (0.76–1.33)
Peripheral vascular disease	1.26 (0.78–2.05)	1.92 (1.40–2.63)^a^	0.81 (0.63–1.04)	1.05 (0.79–1.40)^b^
Renal failure	0.60 (0.37–0.99)^b^	1.80 (1.30–2.48)^a^	0.81 (0.62–1.05)	0.76 (0.56–1.03)
Tumor (without metastasis)	0.95 (0.36–2.51)	1.64 (0.94–2.84)	1.32 (0.84–2.07)	0.75 (0.45–1.26)
**Medications**				
Beta blocker	1.08 (0.65–1.78)	1.32 (0.95–1.83)	1.81 (1.38–2.36)^a^	0.79 (0.59–1.06)
Angiotensin receptor blocker	0.70 (0.65–1.78)	0.93 (0.63–1.38)	1.44 (1.05–1.96)^b^	0.79 (0.56–1.12)
Angiotensin-converting enzyme inhibitor	0.80 (0.43–1.51)	0.93 (0.63–1.38)	1.15 (0.84–1.57)	0.74 (0.52–1.06)
Calcium channel blocker	1.32 (0.66–2.63)	1.32 (0.86–2.03)	0.88 (0.62–1.22)	1.13 (0.77–1.65)
Diuretic	0.52 (0.66–2.63)^b^	0.84 (0.59–1.20)	0.92 (0.69–1.23)	0.73 (0.53–1.00)
Thiazide diuretic	1.35 (0.30–6.22)	0.59 (0.26–1.34)	1.78 (0.85–3.71)	0.54 (0.23–1.25)
Antihypertensive	2.21 (0.94–5.16)	2.22 (1.34–3.69)^b^	1.02 (0.68–1.51)	1.51 (0.96–2.37)
**CDT Composition**				
Physician	2.97 (1.86–4.73)^a^	1.39 (1.01–1.92)^b^	1.01 (0.79–1.30)	0.72 (0.54–0.94)^a^
Resident	1.32 (0.83–2.12)	1.08 (0.79–1.48)	1.23 (0.88–1.44)	1.28 (0.97–1.69)
Advanced nurse practitioner	1.14 (0.71–1.85)	0.86 (0.62–1.17)	1.05 (0.81–1.36)	1.72 (1.30–2.28)^a^
Registered nurse	0.12 (0.04–0.33)^a^	0.50 (0.26–0.98)^b^	1.62 (0.97–2.71)	7.64 (2.93–19.93)^a^
Licensed practical nurse	28421702 (0-∞)	0.79 (0.11–5.51)	0.71 (0.11–4.41)	0.69 (0.09–5.26)
Patient care technician	42.26 (5.62–317.75)^a^	0.73 (0.51–1.06)	0.96 (0.72–1.29)	1.77 (1.27–2.48)^a^
Care manager	119.09 (16.27–871.85)^a^	0.65 (0.48–0.89)^b^	1.20 (0.93–1.53)	2.99 (2.29–3.91^)^^a^
Social worker	180583997 (0-∞)	0.92 (0.65–1.29)	1.10 (0.83–1.44)	1.79 (1.31–2.44)^a^
Physical therapist	165228307665 (0-∞)	0.49 (0.29–0.85)^b^	1.12 (0.72–1.72)	5.12 (3.06–8.57)^a^
Occupational therapist	2630840.79 (0-∞)	1.05 (0.58–1.90)	0.78 (0.49–1.24)	1.09 (0.61–1.95)

^*^Significance:

^a^*P* < .001

^b^*P* < .05’

^**^Odds ratios for unknown race were not provided because they accounted for <1% of encounters and were not statistically significant.

**Table 6:** Binomial logistic regression odds ratios of HFmrEF patients (n = 329).

Variables	Model 1: Hospitalization Odds ratio (95%CI)	Model 2: High number of readmissions Odds ratio (95%CI)	Model 3: High number of days between readmissions Odds ratio (95%CI)	Model 4: High charge encounter Odds ratio (95%CI)
Patient Variables**				
Age	0.48 (0.24–0.98)^b^	0.97 (0.60–1.56)	1.11 (0.74–1.65)	0.83 (0.54–1.28)
Male	0.41 (0.21–0.79)^a^	0.88 (0.56–1.37)	1.23 (0.85–1.78)	1.04 (0.69–1.54)
Female	1	1	1	1
Caucasian American	0	175162041 (0-∞)	2.02 (0.24–17.29)	0
African American	0	132209021 (0-∞)	2.89 (0.34–24.61)	0
Hispanic/Latin/Spanish	3.12 (0-∞)	1012247283 (0-∞)	0.53 (0.02–12.88)	0
Asian American	0.33 (0-∞)	0.30 (0-∞)	2014377860 (0-∞)	1.23 (0-∞)
American Indian/Alaskan Native	0.51 (0-∞)	0.41 (0-∞)	0	0
Native Hawaiian/Other Pacific Islander	0	0	0	0
Other Race	0	0	0	0
**Comorbidities and Medical History**				
Anemia deficiency	0.64 (0.31–0.1.35)	6.94 (4.27–11.30)^a^	0.44 (0.30–0.66)^a^	1.26 (0.80–1.97)
Coagulopathy	1.15 (0.48–2.79)	4.16 (2.32–7.44)^a^	0.56 (0.35–0.88)^b^	1.89 (1.15–3.09)^b^
Diabetes (complicated)	0.48 (0.17–1.40)	1.84 (0.90–3.76)	0.76 (0.43–1.35)	0.87 (0.47–1.63)
Diabetes (uncomplicated)	0.98 (0.36–2.66)	0.64 (0.31–1.31)	1.03 (0.59–1.82)	0.67 (0.37–1.24)
Hypertension (complicated)	27202755(0-∞)	973209690 (0-∞)	0.68 (0.57–8.31)	1.91 (0.04–91.29)
Metastatic cancer	8.71 (0.87–87.17)	2.65 (0.83–8.47)	0.36 (0.14–0.94)^b^	2.52 (0.97–6.57)
Obese	0.91 (0.44–1.90)	2.11 (1.33–3.38)^b^	0.96 (0.65–1.42)	1.11 (0.72–1.70)
Peripheral vascular disease	1.83 (0.89–3.77)	2.07 (1.32–3.27)^b^	0.43 (0.29–0.63)^a^	0.97 (0.64–1.49)
Renal failure	2.08 (0.96–4.49)	2.39 (1.50–3.80)^a^	0.93 (0.63–1.40)	0.60 (0.39–0.94)^b^
Tumor (without metastasis)	3.40 (0.56–20.59)	1.37 (0.62–3.04)	0.58 (0.30–1.13)	1.01 (0.50–2.03)
**Medications**				
Beta blocker	0.97 (0.45–2.08)	3.55 (2.10–5.99)^a^	0.64 (0.42–0.97)^b^	1.16 (0.74–1.82)
Angiotensin receptor blocker	2.46 (0.98–6.22)	3.80 (2.03–7.12)^a^	1.08 (0.66–1.76)	1.01 (0.59–1.73)
Angiotensin-converting enzyme inhibitor	3.57 (1.37–9.31)^b^	1.62 (0.88–2.99)	2.52 (1.52–4.18)^a^	0.71 (0.42–1.20)
Calcium channel blocker	0.53 (0.23–1.21)	1.50 (0.87–2.59)	0.77 (0.49–1.22)	1.18 (0.72–1.93)
Diuretic	1.04 (0.46–2.36)	2.62 (1.51–4.56)^a^	0.86 (0.54–1.35)	1.16 (0.71–1.91)
Thiazide diuretic	0.38 (0.10–1.48)	0.38 (0.15–0.97)^b^	1.14 (0.53–2.47)	0.87 (0.37–2.04)
Antihypertensive	1.09 (0.36–3.32)	0.31 (0.15–0.64)^b^	1.68 (0.92–3.09)	0.56 (0.28–1.10)
**CDT Composition**				
Physician	3.23 (1.53–6.76)^b^	1.33 (0.83–2.14)	0.97 (0.65–1.45)	2.22 (1.44–3.44)^a^
Resident	1.06 (0.51–2.23)	1.19 (0.74–1.91)	1.04 (0.70–1.54)	0.97 (0.63–1.49)
Advanced nurse practitioner	0.28 (0.14–0.59)^a^	0.74 (0.45–1.22)	1.04 (0.69–1.56)	1.12 (0.72–1.75)
Registered nurse	0.16 (0.04–0.68)^b^	0.67 (0.25–1.76)	1.08 (0.49–2.39)	5.75 (1.83–18.07)^b^
Licensed practical nurse	40567798 (0-∞)	2.13 (0.14–31.63)	0.41 (0.35–4.69)	1.81 (0.06–55.14)
Patient care technician	12.02 (2.47–58.45)	1.28 (0.73–2.25)	0.75 (0.47–1.21)	1.20 (0.72–2.02)
Care manager	19 (6.01–60.74)^a^	0.54 (0.34–0.86)^b^	1.70 (1.15–2.53)^b^	2.84 (1.89–4.27)^a^
Social worker	256199996 (0-∞)	0.97 (0.56–1.69)	0.82 (0.51–1.32)	1.85 (1.11–3.08)^b^
Physical therapist	19.40 (2.09–180.08)^b^	0.84 (0.41–1.72)	0.81 (0.44–1.49)	5.74 (2.87–11.48)^a^
Occupational therapist	53297085782 (0-∞)	0.50 (0.23–1.12)	1.03 (0.53–2.01)	0.95 (0.44–2.05)

^*^Significance:

^a^*P* < .001

^b^*P* < .05’

^**^Odds ratios for unknown race were not provided because they accounted for <1% of encounters and were not statistically significant.

**Table 7:** Binomial logistic regression odds ratios of HEpEF patients (n = 707).

Variables	Model 1: Hospitalization Odds ratio (95%CI)	Model 2: High number of readmissions Odds ratio (95%CI)	Model 3: High number of days between readmissions Odds ratio (95%CI)	Model 4: High charge encounter Odds ratio (95%CI)
Patient Variables**				
Age	0.95 (0.75–1.20)	1.14 (0.96–1.35)	0.91 (0.78–1.06)	0.95 (0.80–1.13)
Male	0	1.05 (0.90–1.24)	1.01 (0.87–1.17)	0.99 (0.83–1.17)
Female	0	0	0	0
Caucasian American	0	665205376 (0-∞)	1946289215 (0-∞)	3.93 (0.20–78.99)
African American	0	613754995 (0-∞)	3063189240 (0-∞)	2.07 (0.10–41.63)
Hispanic/Latin/Spanish	0.56 (0.14–2.18)	1.19 (0.54–2.66)	0.60 (0.28–1.27)	0.77 (0.33–1.81)
Asian American	0	498647916 (0-∞)	1005757532 (0-∞)	3.95 (0.15–104)
American Indian/ Alaskan Native	0	1184429949 (0-∞)	3043060563 (0-∞)	1.48 (0.05–41.83)
Native Hawaiian/Other Pacific Islander	37.46 (0-∞)	3.26 (0-∞)	1212349678 (0-∞)	0
Other Race	0	680166065 (0-∞)	1554488424 (0-∞)	0
**Comorbidities and Medical History**				
Anemia deficiency	1	3.11 (2.59–3.74)^a^	0.61 (0.51–0.72)^a^	1.12 (0.92–1.36)
Coagulopathy	1.33 (1–1.77)	2.61 (2.14–3.18)^a^	0.60 (0.50–0.72)^a^	1.65 (1.35–2.01)^a^
Diabetes (complicated)	0.78 (0.55–1.11)	1.18 (0.92–1.51)	0.88 (0.70–1.10)	0.79 (0.61–1.02)
Diabetes (uncomplicated)	1.15 (0.81–1.63)	1.12 (0.86–1.42)	0.91 (0.73–1.14)	1.06 (0.82–1.37)
Hypertension (complicated)	2.11 (0.24–18.13)	0.41 (0.12–1.39)	0.28 (0.06–1.31)	1.37 (0.38–4.86)
Metastatic cancer	1.08 (0.65–1.79)	1.66 (1.14–2.42)^b^	0.43 (0.31–0.61)^a^	1.53 (1.06–2.21)^b^
Obese	0.97 (0.75–1.25)	3.26 (2.73–3.89)^a^	0.87 (0.74–1.02)	0.81 (0.68–0.97)^b^
Peripheral vascular disease	0.97 (0.77–1.23)	1.99 (1.69–2.35)^a^	0.60 (0.52–0.70)^a^	1.12 (0.94–1.34)
Renal failure	1.29 (1.01–1.66)^b^	1.63 (1.37–1.95)^a^	0.59 (0.50–0.70)^a^	1.23 (1.02–1.48)^b^
Tumor (without metastasis)	1.03 (0.73–1.46)	1.67 (1.31–2.13)^a^	0.92 (0.73–1.14)	1.03 (0.80–1.32)
**Medications**				
Beta blocker	0.87 (0.67–1.13)	1.18 (0.98–1.41)	1.01 (0.86–1.20)	1.04 (0.87–1.27)
Angiotensin receptor blocker	0.96 (0.71–1.28)	1.65 (1.32–2.04)^b^	0.80 (0.66–0.97)^b^	0.90 (0.72–1.13)
Angiotensin-converting enzyme inhibitor	1.07 (0.78–1.45)	0.95 (0.76–1.18)	1.19 (0.97–1.46)	0.94 (0.75–1.18)
Calcium channel blocker	0.90 (0.70–1.15)	1.21 (1.02–1.45)^b^	0.90 (0.76–1.05)	0.97 (0.80–1.16)
Diuretic	1.13 (0.86–1.47)	0.90 (0.75–1.08)	1.20 (1.02–1.42)^b^	0.88 (0.72–1.06)
Thiazide diuretic	1.21 (0.76–1.91)	0.51 (0.37–0.71)^a^	1.09 (0.81–1.47)	1.19 (0.85–1.66)
Antihypertensive	1.30 (0.89–1.90)	1.43 (1.09–1.87)^b^	1.24 (0.97–1.59)	1.01 (0.76–1.33)
**CDT Composition**				
Physician	2.07 (1.61–2.66)^a^	0.97 (0.81–1.15)	1.04 (0.89–1.22)	1.93 (1.62–2.31)^a^
Resident	1.05 (0.82–1.36)	0.95 (0.79–1.13)	1.34 (1.15–1.57)^a^	1.22 (1.02–1.46)^b^
Advanced nurse practitioner	0.45 (0.35–0.58)^a^	0.98 (0.82–1.17)	0.97 (0.83–1.14)	1.57 (1.30–1.88)^a^
Registered nurse	0.15 (0.09–0.24)^a^	0.48 (0.34–0.68)^a^	1.47 (1.09–1.98)^b^	3.33 (2.20–5.04)^a^
Licensed practical nurse	60040903 (0-∞)	0.89 (0.29–2.69)	2.55 (0.86–7.60)	0.72 (0.24–2.10)
Patient care technician	30.96 (12.53–76.51)^a^	0.75 (0.61–0.91)^b^	0.92 (0.76–1.10)	2.63 (2.12–3.27)^a^
Care manager	7.81 (5.42–11.26)^a^	0.82 (0.69–0.98)^b^	0.96 (0.82–1.13)	2.09 (1.75–2.49)^a^
Social worker	243679512 (0-∞)	0.76 (0.61–0.93)^b^	1.10 (0.91–1.33)	1.89 (1.52–2.36)^a^
Physical therapist	27.45 (9.45–79.78)^a^	0.85 (0.65–1.11)	1.02 (0.79–1.30)	4.99 (3.71–6.70)^a^
Occupational therapist	3.30 (0.77–14.13)	0.70 (0.53–0.94)^b^	0.95 (0.73–1.24)	1.43 (1.02–2.01)^b^

^*^Significance:

^a^*P* < .001

^b^*P* < .05’

^**^Odds ratios for unknown race were not provided because they accounted for <1% of encounters and were not statistically significant.

Model 1 of Table 4 reflects CHF patient hospitalization risk (i.e., being admitted to the hospital without an admission within the preceding 30 days) over the 7-year study period. Hospitalizations for all CHF patients (i.e., 5,962) had a mean of 27.67, a median of 25.72, and a mode of 48.13. The model explained 64.2% (Nagelkerke R^2^) [50] of the variance in hospitalizations and correctly classified 85.2% of cases. CHF patients who were AA were 84% less likely (i.e., Odds Ratio = 0.16; Table 4) to be hospitalized when compared to all non-AAs. CHF patients with a Registered Nurse on their CDT were 30% less likely to have a hospitalization. However, CHF patients with other clinical roles on their CDTs were more likely to have a hospitalization: a Physician (i.e., 3.02 times more likely), Advanced Nurse Practitioner (i.e., 1.12 times more likely), Patient Care Technician (i.e., 29.43 times more likely), Care Manager (i.e., 21.87 times more likely), Social Worker (i.e., 1,491 times more likely), Physical Therapist (i.e., 216.18 times more likely), and an Occupational Therapist (i.e., 7.30 times more likely).

Model 2 of Table 4 reflects CHF patient readmission risk (i.e., being admitted to the hospital within 30 days of a discharge) over the 7-year study period. Readmissions for all patients had a mean of 51.87, a median of 48, and a mode of 96. The model explained 49.4% of the variance in readmissions and correctly classified 78.0% of cases. Over the 7-year study period, male CHF patients were 11% more likely to have a high number of readmissions. CHF patients who were AA were 11.28 times more likely to have a high number of readmissions when compared to all non-AAs. CHF patients with a Physician or a Resident on their CDTs were 13% less likely to have a high number of readmissions. In addition, CHF patients with other clinical roles on their CDTs were less likely to have a high number of readmissions: a Registered Nurse (i.e., 31%), Licensed Practical Nurse (i.e., 40%), Patient Care Technician (i.e., 19%), Care Manager (i.e., 19%), Social Worker (i.e., 17%), Physical Therapist (i.e., 28%), and an Occupational Therapist (i.e., 14%).

Model 3 of Table 4 reflects CHF patients who were at risk for a high number of days between readmissions (i.e., more days at home) over the 7-year study period. For days between readmissions, all CHF patients had a mean of 27.67, a median of 25.72, and a mode of 48.13. The model explained 22.2% of the variance in the number of days between readmissions and correctly classified 67.4% of cases. Over the 7-year study period, CHF patients above age 59 (i.e., the median) were 9% more likely to have a high number of days between readmissions. However, male CHF patients were 13% less likely to have a high number of days between readmissions. AA patients were 7.84 times more likely to have a high number of days between readmissions when compared to all non-AAs. CHF patients with a Resident or a Licensed Practical Nurse on their CDTs were 11% and 37% more likely to have a high number of days between readmissions, respectively. In addition, CHF patients with other clinical roles on their CDTs were less likely to have a high number of days between readmissions (i.e., less days at home): an Advanced Nurse Practitioner (i.e., 11%), Registered Nurse (i.e., 8%), Patient Care Technician (i.e., 8%), Social Worker (i.e., 12%), and a Physical Therapist (i.e., 2%).

Model 4 of Table 4 reflects CHF patient risk for a high charge encounter over the 7-year study period. For all patients, care encounter charges had a mean of $37,801, a median of $22,403, and a mode of $91,208. The model explained 45.2% of the variance in charges per encounter and correctly classified 77.1% of cases. Over the 7-year study period, CHF patients who were AA were 66% less likely to have a high charge care encounter (i.e., charge above the median of $22,403) when compared to all non-AAs. CHF patients with a Licensed Practical Nurse on their CDT were 12% less likely to have a high charge care encounter. In addition, CHF patients with other clinical roles on their CDTs were more likely to have a high charge encounter: a Physician (i.e., 2.59 times more likely), Resident (i.e., 30%), Advanced Nurse Practitioner (i.e., 79%), Registered Nurse (i.e., 6%), Patient Care Technician (i.e., 3.77 times more likely), Care Manager (i.e., 4.84 times more likely), Social Worker (i.e., 3.32 times more likely), Physical Therapist (i.e., 8.82 times more likely), and an Occupational Therapist (i.e., 32%).

### Results stratified by heart failure severity

#### Heart failure patients with reduced ejection fraction

We categorized left ventricular ejection fraction (i.e., the amount of blood in the chamber compared to the amount of blood pumped out of the chamber) into heart failure phenotypes consistent with the New York Heart Association Classification and practice guidelines [51–54]. There were 628 patients with ejection fraction of less than or equal to 40 percent (Table 5), classified as heart failure with *reduced* ejection fraction (i.e., HFrEF) which indicated patients with the most severe cases of heart failure [51, 52, 54].

When stratified by heart failure phenotype, Model 1 of Table 5 reflects HFrEF patient hospitalization risk (i.e., being admitted to the hospital without an admission within the preceding 30 days) over the 7-year study period. Hospitalizations for HFrEF patients (n = 628) had a mean of 42.90, a median of 35, and a mode of 96. The model explained 54.9% of the variance in hospitalizations and correctly classified 90.9% of cases. HFrEF patients (i.e., the most severe heart failure cases) with renal failure or on a diuretic were 40% and 48% less likely to have a hospitalization, respectively. Notably, HFrEF patients with a registered nurse on their CDT were 88% less likely to have a hospitalization. However, HFrEF patients with other clinical roles on their CDTs were more likely to have a hospitalization: a physician (i.e., 2.97 times more likely), patient care technician (i.e., 42.26 times more likely), and a care manager (i.e., 119.09 times more likely).

Model 2 of Table 5 reflects HFrEF patient readmission risk (i.e., being admitted to the hospital within 30 days of a discharge) over the 7-year study period. Readmissions for HFrEF patients had a mean of 8.08, a median of 2, and a mode of 1. The model explained 55.8% of the variance in readmissions and correctly classified 81.1% of cases. In readmissions, HFrEF patients with specific clinical roles on their CDTs were less likely to have a high number of readmissions: a registered nurse (i.e., 50%), care manager (i.e., 35%), and a physical therapist (i.e., 51%). However, HFrEF patients were more likely to have a high number of readmissions if they had anemia deficiency (i.e., 93%), complicated diabetes (i.e., 2.91 times more likely), metastatic cancer (i.e., 5.66 times more likely), obesity (i.e., 2.39 times more likely), peripheral vascular disease (i.e., 92%), renal failure (i.e., 80%), and were on an antihypertensive (i.e., 2.22 times more likely). Additionally, HFrEF patients were 39% more likely to have a high number of readmissions if they had a physician on their CDT.

Model 3 of Table 5 reflects HFrEF patient risk of having a high number of days between readmissions (i.e., more days at home) over the 7-year study period. Days between readmissions for HFrEF patients had a mean of 31.13, a median of 31.67, and a mode of 48.13. The model explained 20.4% of the variance in the number of days between readmissions and correctly classified 68.3% of cases. HFrEF patients were more likely to have a high number of days between readmissions (i.e., more days at home) if they were on a beta blocker (i.e., 81%) and an angiotensin receptor blocker (i.e., 44%). HFrEF patients were less likely to have a high number of days between readmissions (i.e., less days at home) if they had anemia deficiency (i.e., 29%) and uncomplicated diabetes (i.e., 32%).

Model 4 of Table 5 reflects HFrEF patient risk for a high charge encounter over the 7-year study period. Care encounter charges for HFrEF patients had a mean of $54,273.69, a median of $54,303.37, and a mode of $91,208. The model explained 20.4% of the variance in charges per care encounter and correctly classified 68.3% of cases. In care encounter charges, HFrEF patients with a physician on their CDTs were 28% less likely to have a high charge care encounter. However, HFrEF patients with other clinical roles on their CDT were more likely to have a high charge care encounter: an advanced nurse practitioner (i.e., 72%), registered nurse (i.e., 7.64 times more likely), patient care technician (i.e., 77%), care manager (i.e., 2.99 times more likely), social worker (i.e., 79%), and a physical therapist (i.e., 5.12 times more likely).

#### Heart failure patients with mildly reduced ejection fraction

There were 329 patients with ejection fraction of greater than 40% but less than 50% (Table 6), classified as heart failure with *mildly reduced* ejection fraction (i.e., HFmrEF) which indicated patients with intermediate heart failure severity [51, 52, 54].

Table 6 reflects HFmrEF patients, a heart failure phenotype representing patients with intermediate cases of heart failure. Model 1 of Table 6 reflects HFmrEF patient hospitalization risk (i.e., being admitted to the hospital without an admission within the preceding 30 days) over the 7-year study period. Hospitalizations for HFmrEF patients (n = 329) had a mean of 49.45, a median of 47, and a mode of 96. The model explained 57.8% of the variance in hospitalizations and correctly classified 69.5% of cases. HFmrEF patients who were male and above age 64 (i.e., the median) were 59% and 52% less likely to have a hospitalization, respectively. Additionally, HFmrEF patients with an advanced nurse registered nurse or a registered nurse on their CDT were 72% and 84% less likely to have a hospitalization, respectively. However, HFmrEF patients with other clinical roles on their CDTs were more likely to have a hospitalization: a physician (i.e., 3.23 times more likely), care manager (i.e., 19 times more likely), and a physical therapist (i.e., 119.09 times more likely). HFmrEF patients who were on a angiotensin-converting enzyme inhibitor were also 3.57 times more likely to have a hospitalization.

Model 2 of Table 6 reflects HFmrEF patient readmission risk (i.e., being admitted to the hospital within 30 days of a discharge) over the 7-year study period. Readmissions for HFmrEF patients had a mean of 7.93, a median of 2, and a mode of 1. The model explained 54.4% of the variance in readmissions and correctly classified 79.5% of cases. HFmrEF patients with a care manager on their CDTs were 46% less likely to have a high number of readmissions. HFmrEF patients who were on a thiazide diuretic and an antihypertensive were 62% and 69% less likely to have a high number of readmissions. However, HFmrEF patients were more likely to have a high number of readmissions if they had anemia deficiency (i.e., 6.94 times more likely), coagulopathy (i.e., 4.16 times more likely), obesity (i.e., 2.11 times more likely), peripheral vascular disease (i.e., 2.07 times more likely), and renal failure (i.e., 2.39 times more likely). In addition, HFmrEF patients were more likely to have a high number of readmissions if they were on a beta blocker (i.e., 3.55 times more likely), an angiotensin receptor blocker (i.e., 3.80 times more likely), or a diuretic (i.e., 2.62 times more likely).

Model 3 of Table 6 reflects HFmrEF patient risk of having a high number of days between readmissions (i.e., more days at home) over the 7-year study period. Days between readmissions for HFmrEF patients had a mean of 31.02, a median of 31.87, and a mode of 48.13. The model explained 26.8% of the variance in the number of days between readmissions and correctly classified 68.3% of cases. HFmrEF patients were more likely to have a high number of days between readmissions (i.e., more days at home) if they were on an angiotensin-converting enzyme inhibitor (i.e., 2.52 times more likely) or had a social worker on their CDT (i.e., 70%). HFmrEF patients were less likely to have a high number of days between readmissions (i.e., less days at home) if they had anemia deficiency (i.e., 56%), coagulopathy (i.e., 44%), metastatic cancer (i.e., 64%), or peripheral vascular disease (i.e., 57%).

Model 4 of Table 6 reflects HFmrEF patient risk for a high charge encounter over the 7-year study period. Care encounter charges for HFmrEF patients had a mean of $55,382.62, a median of $55,544.67, and a mode of $91,208. The model explained 40.2% of the variance in charges per care encounter and correctly classified 74.7% of cases. HFmrEF patients with renal failure were 40% less likely to have a high charge care encounter. However, HFmrEF patients with specific clinical roles on their CDT were more likely to have a high charge care encounter: a physician (i.e., 2.22 times more likely), registered nurse (i.e., 5.75 times more likely), care manager (i.e., 2.84 times more likely), social worker (i.e., 85%), or a physical therapist (i.e., 5.74 times more likely).

#### Heart failure patients with preserved ejection fraction

There were 707 patients with ejection fraction of greater than or equal to 50 percent (Table 7), classified as heart failure with *preserved* ejection fraction (i.e., HFpEF) which indicated patients with the least severe cases of heart failure [51, 52, 54].

Table 7 reflects HFpEF patients, a heart failure phenotype representing patients with the least severe cases (i.e., near normal pumping) of heart failure. Model 1 of Table 7 reflects HFpEF patient hospitalization risk (i.e., being admitted to the hospital without an admission within the preceding 30 days) over the 7-year study period. Hospitalizations for HFpEF patients (n = 707) had a mean of 53.61, a median of 51, and a mode of 96. The model explained 50.7% of the variance in hospitalizations and correctly classified 84.9% of cases. Additionally, HFpEF patients with an advanced practice registered nurse or a registered nurse on their CDT were 55% and 85% less likely to have a hospitalization, respectively. However, HFpEF patients with specific clinical roles on their CDTs were more likely to have a hospitalization: a physician (i.e., 2.07 times more likely), care manager (i.e., 7.81 times more likely), and a physical therapist (i.e., 27.45 times more likely). Additionally, HFpEF patients with renal failure were also 29% more likely to have a hospitalization.

Model 2 of Table 7 reflects HFpEF patient readmission risk (i.e., being admitted to the hospital within 30 days of a discharge) over the 7-year study period. Readmissions for HFpEF patients had a mean of 7.81, a median of 2, and a mode of 0. The model explained 36.5% of the variance in readmissions and correctly classified 72.2% of cases. HFpEF patients with specific clinical roles on their CDT were less likely to have a high number of readmissions: a registered nurse (i.e., 52%), patient care technician (i.e., 25%), care manager (i.e., 18%), social worker (i.e., 24%), or an occupational therapist (i.e., 30%). HFpEF patients who were on a thiazide diuretic were 49% less likely to have a high number of readmissions. However, HFpEF patients were more likely to have a high number of readmissions if they had anemia deficiency (i.e., 3.11 times more likely), coagulopathy (i.e., 2.61 times more likely), metastatic cancer (i.e., 66%), obesity (i.e., 3.26 times more likely), peripheral vascular disease (i.e., 99%), renal failure (i.e., 63%), or a tumor without metastasis (i.e., 67%). In addition, HFpEF patients were more likely to have a high number of readmissions if they were on an angiotensin receptor blocker (i.e., 65%), a calcium channel blocker (i.e., 21%), or an antihypertensive (i.e., 43%).

Model 3 of Table 7 reflects HFpEF patient risk of having a high number of days between readmissions (i.e., more days at home) over the 7-year study period. Days between readmissions for HFpEF patients had a mean of 30.01, a median of 29.35, and a mode of 48.13. The model explained 17% of the variance in the number of days between readmissions and correctly classified 65.6% of cases. HFpEF patients were more likely to have a high number of days between readmissions (i.e., more days at home) if they were on a diuretic (i.e., 20%), or had a resident (i.e., 34%) or registered nurse (i.e., 47%) on their CDT. HFpEF patients were less likely to have a high number of days between readmissions (i.e., less days at home) if they had anemia deficiency (i.e., 39%), coagulopathy (i.e., 40%), metastatic cancer (i.e., 57%), peripheral vascular disease (i.e., 40%), renal failure (i.e., 41%), or if they were on an angiotensin receptor blocker (i.e., 20%).

Model 4 of Table 7 reflects HFpEF patient risk for a high charge encounter over the 7-year study period. Care encounter charges for HFpEF patients had a mean of $52,835.96, a median of $52,892.97, and a mode of $91,208. The model explained 41.6% of the variance in charges per care encounter and correctly classified 76.1% of cases. HFpEF patients were less likely to have a high charge care encounter if they had metastatic cancer (i.e., 19%). However, HFpEF patients with specific comorbidities and medical histories were more likely to have a high charge care encounter: coagulopathy (i.e., 65%), metastatic cancer (i.e., 53%), or renal failure (i.e., 23%). HFpEF patients with specific clinical roles on their CDT were more likely to have a high charge care encounter: a physician (i.e., 93%), resident (i.e., 22%), advanced nurse practitioner (i.e., 57%), registered nurse (i.e., 3.33 times more likely), patient care technician (i.e., 2.63 times more likely), care manager (i.e., 2.09 times more likely), social worker (i.e., 89%), physical therapist (i.e., 4.99 times more likely), or an occupational therapist (i.e., 43%).

**Fig 4:**
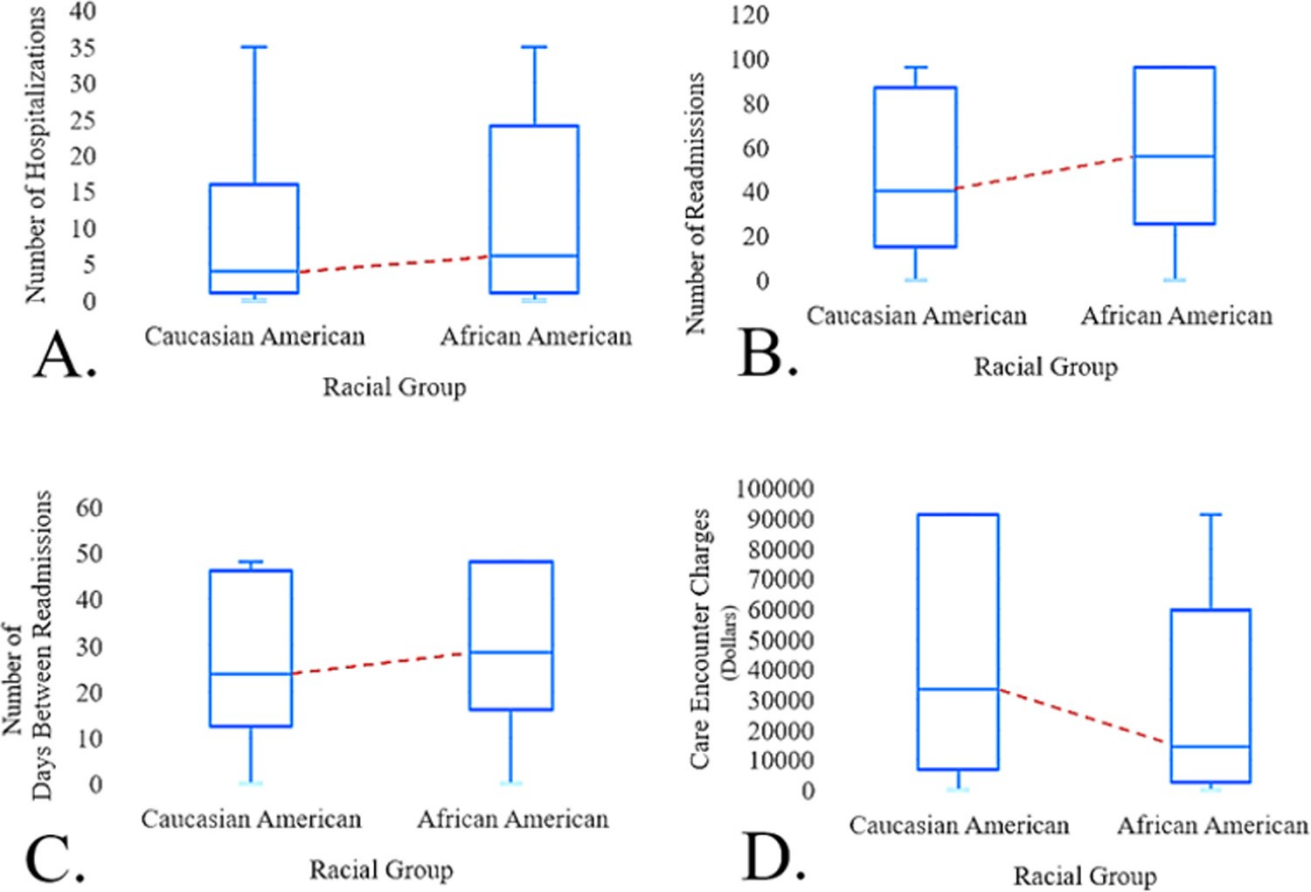
Mann Whitney-U median differences between CAs and AAs.

Furthermore, Fig 4 illustrates median differences between AAs (n = 39,076) and CAs (n = 39,187) in total care encounters, each group accounting for approximately 48% of total care encounters. As demonstrated by Fig 4A, AAs had a significantly higher number of hospitalizations (*p* = .001) than CAs (i.e., 6 *vs*. 4, respectively). As a group, AAs had a significantly higher number of readmissions (*p* = .001) than CAs (i.e., 56 *vs*. 40, respectively; Fig 4B). In addition, AAs had a significantly higher number of days between readmissions [days at home] (*p* = .001) than CAs (i.e., 28.34 *vs*. 23.70, respectively; Fig 4C). However, AAs had significantly lower care encounter charges (*p* = .001) than CAs (i.e., $14,249.22 *v*. $33,213.78, respectively; Fig 4D).

## Discussion

### CDT composition influence on care outcomes

The EMR is the ecosystem for care delivery, providing wide-ranging and longitudinal data and opportunities for stratifying patient risk of poor outcomes [38]. Recent studies have called for EMR-driven examinations of the optimal CDT composition (i.e., size, roles, structures, and interaction) to support high-quality personalized care [22, 23, 38]. The most widely accepted model of CDT composition is the Veterans Health Administration’s Patient Aligned Care Teams model [39]. While fundamental to the domain, variation exists in how primary care clinics have operationalized the model─ lacking specificity in expressing which specific CDT clinical role are highly associated with patient risk of hospitalization, readmission, and high charge encounters [22, 39]. Our findings suggested that CHF patients may have benefited the most from the care of registered nurses more than any other CDT role. Notably, the registered nurse was the most heavily engaged clinical role found among care delivery encounters of CHF patients. Among all patients, registered nurses were the only CDT role consistently associated with a 30% decreased risk of hospitalization and a 31% decrease in risk of having a high number of readmissions during the 7-year study period. In the most severe heart failure cases, HFrEF patients with a registered nurse on their CDT were 88% less likely to have hospitalization and were 50% less likely to have a high number of readmissions. Similar decreases in risk of hospitalization and readmission were also found in the less severe cases of heart failure (i.e., HFmrEF and HFpEF). However, the possible reasons for the association between registered nurses and improved care outcomes have been thoroughly evaluated in other studies. The key role of the registered nurse in the management of CHF is largely focused on patient monitoring (i.e., early intervention, medication management, outpatient follow-up, self-care education, and care coordination) [55]. Registered nurses are also responsible for initial assessment and triage, discharge planning, and end-of-life care [55–57].

However, there is variation in the scope of practice among registered nurses, influenced by various factors (e.g., educational level of the nurse, state of practice, and clinical environment and resources). However, registered nurses generally educate patients on the causes of CHF and facilitate discussions on lifestyle modifications (e.g., eliminating/reducing sodium intake and increasing physical activity). With responsibilities that require such high levels of engagement with patients, registered nurses also act as an early alert system, creating more complex and compounded care when required for high-need CHF patients. For example, a registered nurse’s triage results (e.g., abnormal electrocardiogram read) could indicate the need to immediately engage more CDT members (e.g., the code team or cardiologist). The findings affirm that registered nurses are the most impactful (i.e., highly associated with positive patient outcomes) frontline providers. The findings also emphasize the value of the responsibilities undertaken by registered nurses (e.g., managing CHF risk factors, symptom management, and education and counseling). These responsibilities help explain why registered nurses were the most frequent clinical role found among care delivery encounters and solidify their role in the development and implementation of future interventions that target decreasing hospitalizations in CHF patients.

Decreasing hospitalizations in AA patients is particularly impactful to CHF patients because heart failure-related hospitalizations for AA patients [both men and women] are nearly 2.5 times higher than CA patients, nationally [58–61]. Yet, the CDTs of CHF patients generally include a diverse set of care types and clinical roles [41–43]. Research suggests patients benefit from the composite of different types of clinical training offered by the variation and unique contributions of each clinical role to the patient’s care plan [41–43]. The only known study in the domain evaluated associations between primary care team communication, interaction, and costs for patients with cardiovascular disease in 155 CDT members (e.g., physicians, advanced nurse practitioners, registered nurses) at 6 primary care clinics [18]. The study found that CDTs that were more interconnected, less centralized, and had a shared team vision were more likely to deliver high quality cardiovascular care at a lower cost than CDTs who were not [18]. However, the study did not investigate the extent to which specific clinical roles were associated with improving hospitalizations or care costs. Studies have also been conducted on other chronic diseases, posing similar questions and yielding similar results [62, 63]. Even with the additional contribution of this study, the contributions of all clinical roles can not be minimized or devalued because the literature related to CDT composition is in its infancy. However, this finding stimulates the need for future research investigating the role of specific clinical roles in designing, risk stratifying, contributing to, and operationalizing the care plans of AA patients with CHF. Additional evidence will advance the framework for determining if patients have “access to high-quality care” by requiring patients to have access to the clinical roles that are associated with improved care outcomes in specific diseases. Correspondingly; as a recommendation for change in clinical practice, increasing the number of registered nurses on CDTs of AA patients with CHF may decrease hospitalizations and increase days between readmissions. In addition, hospitals and clinics that provide care for large populations of African Americans should consider optimizing the role of the registered nurse by ensuring that registered nurses are more specialized in CHF care delivery and prepared to trigger early alert systems within their organization’s current risk-based care guidelines.

### Implications for population health management

While the study design and results could not determine that CDT composition caused better or worse outcomes for CHF patients, the results emphasized how heavily associated clinical roles are with a CHF patient’s risk of poor or improved care outcomes. For example, among all patients, CHF patients with a physician on their CDT were 3.02 times more likely to have a hospitalization during the 7-year period. When stratified by heart failure severity, the findings on hospitalization risk were relatively consistent among HFrEF (i.e., 2.97 times more likely), HFmrEF (i.e., 3.23 times more likely), and HFpEF patients (i.e., 2.07 times more likely). CHF patients with a resident on their CDTs were 11% more likely to have a high number of days between readmissions (i.e., more days at home) and 30% more likely to have a high charge encounter, but 13% less likely to have a high number of readmissions. These results do not indicate that physicians and residents caused poor or better outcomes; rather, that they were associated with the outcomes. More specifically, these results indicate that these physicians and residents followed a model of care delivery that required them to heavily engage with high-need patients (i.e., physicians and residents are generally more heavily associated with the sickest patients). However, as evidence of this, we would have expected to observe significant differences between HFrEF and HFpEF patients in hospitalizations and readmissions. Yet, the results did not reveal any statistically significant indication of this. One of the most prevalent and recent models of care delivery is the Comprehensive Care Physician Model, where physicians focus on providing care to patients who are at an increased risk of hospitalization, simultaneously supporting inpatient and outpatient care [57]. This care model was an evolution of the Traditional Model of care delivery (i.e., 1990’s) in which risk of hospitalization was not used to evaluate the extent to which physicians [or residents] should have engaged in the care of high-risk patients [64].

Risk stratification (RS), the core of population health management, is nationally recognized as the clinician-driven process of categorizing patients into tiers based on health and social factors to reflect the potential for unplanned healthcare utilization and expensive encounters [13]. In primary care settings [the gateway for all other specialties], processes for RS are relatively new [16, 18]. Among consistently applied RS approaches, the process of tiering patients (i.e., highly-complex, high-risk, rising-risk, low-risk) involves three sequential steps; characterizing the patient population, systematically assigning risk levels, and mitigating risk [13, 16, 26–32]. Systematically assigning risk levels fuels the planning, development, and implementation of personalized care plans, and is the most critical component of population health management [31, 32]. The "one-size-fits-all" approach to care planning and delivery (i.e., same level of resources provided to all patients) is clinically ineffective and wasteful [32]. Assigning and prioritizing individual patients’ needs based on risks promotes the efficient allocation of clinical resources (i.e., clinical team), reserving high-intensity resources for patients who are highly-complex and high-need [32]. Of the current RS approaches, computational tools are employed; yet, clinical intuition (i.e, experience-based, decision-making) is the most widely-used practice [29–31]. Consequently, the subjectivity in how CDTs employ their clinical intuition may promote disparities in the outcomes of cardiovascular disease patients (i.e., women, AAs, elderly) [29–31]. Thus, clinical intuition, alone, is not a consistent, sufficient, and comprehensive RS approach. RS can be approached more comprehensively by working in concert with computational tools, constituting the fundamental theorem of biomedical informatics [33]. Current computational algorithms for CHF include the: Adjusted Clinical Groups, Charlson Comorbidity, Elixhauser Comorbidity Indices, Hierarchical Condition Categories, and Framingham Risk Score [34–36]. Broadly, current tools rely on aggregated patient demographic data (e.g., age, sex, race, marital status), comorbidities (e.g., number and type of chronic conditions), patient panels (e.g., glucose, lipid, systolic and diastolic levels), and behavioral (e.g., diet, physical activity, smoking) to compute a single risk score that assigns patients into risk tiers [16, 17, 34–36]. However, in solely relying on such indicators [ignoring care delivery influences], current tools have not optimized their utility in comprehensively approaching RS [13, 28, 37].

The statistically significant CDT findings suggest that current tools could be expanded to support clinical staffing needs. Hospitals, clinics, and community health centers could improve clinical efficiency with computational tools that can accurately predict how allocating specific clinical roles to patients with the greatest needs would impact and potentially improve care outcomes. More broadly, now that we know that CDT composition is associated with care outcomes, CDT variables can be built into EMRs and data warehouses as a real-time, hospital-level surveillance tool for stratifying patient risk of poor outcomes.

### Limitations

The study data was generated from Arkansas’ only academic health center. While the data reflects patient encounters throughout Arkansas (i.e., 30 of UAMS’ regional clinics in Northeast, Northwest, Central, Southeast, and Southwest Arkansas), findings may not be generalizable to healthcare systems throughout the United States. The lack of heart failure phenotype data on 4,298 of the study’s patients may explain the large mean differences in hospitalizations between entire study population (Table 4) and the subgroups when the analysis was stratified (Tables 5–7). The lack of data may also have impacted the results of the odds ratios in Tables 5–7. However, confidence intervals are provided to approximate any potential variation. Furthermore, the use of heart failure phenotype, alone, did not provide a comprehensive indication of heart failure severity. Consistent with the wide-recognized use of the New York Heart Association Classification, both objective and subjective assessments of heart failure severity would have provided a stronger indication of severity. Clinician-driven, subjective assessments (e.g., observations of shortness of breath, tiredness, and lack of appetite) and other pseudo indicators (e.g., functional status, frailty, and oxygen support needs) were not available to support this study. While this study found CDT roles were statistically significant predictors of care outcomes, larger-scale studies are needed to assess the effectiveness of expanding the current set of risk factors to include CDT composition as a predictor of patient hospitalizations, readmissions, and charges. This study also limited its inquiry to specific clinical roles and did not comprehensively assess associations between outcome variables and all possible roles which contributed to the care of CHF patients. Furthermore, we recognize that cardiologists and other specialty physicians have a vital role in caring for CHF patients. However, the data infrastructure of the AR-CDR aggregated all types of physicians into the role of “physician”. Therefore, our results did not distinguish between generalist and specialty physicians (i.e., cardiologist). Finally, a potential constraint of future research will be the lack of access to more national, EMR data that can match CHF patients with their CDTs. Therefore, it is further recommended that cardiovascular clinical data registries expand by adding CDT-specific data elements (i.e., de-identified CDT numbers, size, structure, and interactions among other CDT) that will more globally support clinical and clinical research studies in the domain. Provisionally, additional smaller-scale, healthcare system-level studies of CDT will help expand the domain.

## Conclusions

The work presented here provides evidence to support the notion that specific clinical roles within CDTs can impact CHF care outcomes for CHF patients, more specifically, those who are disproportionally impacted by CHF. Notably, CDTs comprised of registered nurses resulted in more positive outcomes (i.e., reduced hospitalizations and readmissions), even in patients with the most severe cases of heart failure. These results are the foundation for the underexplored area of care delivery team reformation in which future advances can be used to improve care outcomes and reduce healthcare utilization of patients with CHF. Intentionality must be assumed in the composition of the CDT to improve the planning and delivery of care. Additional research is needed to build, evaluate, and test the efficacy of empirical models of care delivery team composition.
